# l-Arabinose improves hypercholesterolemia via regulating bile acid metabolism in high-fat-high-sucrose diet-fed mice

**DOI:** 10.1186/s12986-022-00662-8

**Published:** 2022-04-15

**Authors:** Yu Wang, Jiajia Zhao, Qiang Li, Jinxin Liu, Yujie Sun, Kuiliang Zhang, Mingcong Fan, Haifeng Qian, Yan Li, Li Wang

**Affiliations:** 1 State Key Laboratory of Food Science and Technology, School of Food Science and Technology, Jiangnan University, Wuxi, 214122 China; 2 College of Cooking Science and Technology, Jiangsu College of Tourism, Yangzhou, 225000 China; 3 China National Institute of Standardization, No. 4 Zhichun Road, Haidian District, Beijing, China

**Keywords:** l-Arabinose, Cholesterol metabolism, Bile acid, Hypercholesterolemia

## Abstract

**Background:**

Hypercholesterolemia is closely associated with an increased risk of cardiovascular diseases. l-Arabinose exhibited hypocholesterolemia properties, but underlying mechanisms have not been sufficiently investigated. This study aimed to elucidate the mechanisms of l-arabinose on hypocholesterolemia involving the enterohepatic circulation of bile acids.

**Methods:**

Thirty six-week-old male mice were randomly divided into three groups: the control group and the high-fat-high-sucrose diet (HFHSD)-fed group were gavaged with distilled water, and the l-arabinose-treated group were fed HFHSD and received 400 mg/kg/day l-arabinose for 12 weeks. Serum and liver biochemical parameters, serum and fecal bile acid, cholesterol and bile acid metabolism-related gene and protein expressions in the liver and small intestine were analyzed.

**Results:**

l-Arabinose supplementation significantly reduced body weight gain, lowered circulating low-density lipoprotein cholesterol (LDL-C) while increasing high-density lipoprotein cholesterol (HDL-C) levels, and efficiently alleviated hepatic inflammation and lipid accumulations in HFHSD-fed mice. l-Arabinose inhibited cholesterol synthesis via downregulation of 3-hydroxy-3-methylglutaryl-CoA reductase (HMGCR). Additionally, l-arabinose might facilitate reverse cholesterol transport, evidenced by the increased mRNA expressions of low-density lipoprotein receptor (LDL-R) and scavenger receptor class B type 1 (SR-B1). Furthermore, l-arabinose modulated ileal reabsorption of bile acids mainly through downregulation of ileal bile acid-binding protein (I-BABP) and apical sodium-dependent bile acid transporter (ASBT), resulting in the promotion of hepatic synthesis of bile acids via upregulation of cholesterol-7α-hydroxylase (CYP7A1).

**Conclusions:**

l-Arabinose supplementation exhibits hypocholesterolemic effects in HFHSD-fed mice primarily due to regulation of bile acid metabolism-related pathways.

**Supplementary Information:**

The online version contains supplementary material available at 10.1186/s12986-022-00662-8.

## Introduction

Cardiovascular diseases (CVD) are the leading determinant of death worldwide, and abnormal cholesterol metabolism is strongly associated with an elevated risk of CVD [1]. Along with unhealthy dietary habits and lifestyles, such as excessive intake of high-fat-high-sucrose diet (HFHSD), the prevalence of hypercholesterolemia increases year by year [2]. Epidemiological evidence has demonstrated that ascending levels of total cholesterol (TC) and low-density lipoprotein cholesterol (LDL-C) contribute to the development of CVD, while the high-density lipoprotein cholesterol (HDL-C) is related to decreased risk for CVD events [3, 4]. Thus, amelioration or reversion of the progression of hypercholesterolemia might be the target of CVD prevention. However, prolonged administration of drugs for hypercholesterolemia may induce undesirable side effects [3]. Therefore, dietary intake of cholesterol-lowering natural products has been considered as potential candidates suitable for patients with low or moderate hypercholesterolemia.

l-Arabinose, an aldopentose in plants, usually extracted from corn cobs, beet pulp, or wheat bran, has been proved to possess benefits in alleviating lipid metabolic disorder, improving insulin resistance, and anti-inflammation [5, 6]. Previous studies have demonstrated that l-arabinose effectively alleviates hyperlipidemia caused by the high consumption of dietary fat and sucrose [7]. For instance, l-arabinose treatment showed cholesterol-lowering properties according to lowering triglyceride (TG), TC, and LDL-C levels and increasing HDL-C levels in metabolic syndrome rats induced by a high-carbohydrate, high-fat (HCHF) diet [8]. Recently, it was reported that l-arabinose could alleviate high-fat-diet-induced metabolic syndrome in mice by modulating the expression of genes governing lipid metabolism and mitochondrial function, effectively restoring altered lipid profile both in the serum and liver [9]. Furthermore, the major mechanism underlying l-arabinose’s hypolipidemic effects may be attributed to inhibition of intestinal sucrase activity, thereby delaying sucrose utilization, and consequently reducing lipogenesis [7, 10, 11]. Although the intervention of l-arabinose exhibited cholesterol-lowering effects, the underlying mechanisms remain to be further investigated.

Maintaining cholesterol homeostasis is crucial for metabolic health. Cholesterol metabolism comprises a tightly regulated process of cholesterol biosynthesis, absorption, transport, and catabolism, which involves diverse transporters, enzymes, and receptor proteins [12, 13]. Both inhibition of cholesterol synthesis in the liver and acceleration of reverse transport from peripheral tissue to liver, representing major hypocholesterolemic mechanisms, are beneficial to lowering circulating cholesterol levels [14, 15]. Since cholesterol is the precursor to bile acid synthesis in the liver, no doubt regulating cholesterol homeostasis is influenced profoundly by bile acid metabolism [16]. The enterohepatic circulation of bile acids contains a complex network of hepatic bile acid synthesis and excretion, intestinal reabsorption, and transport to the liver [17]. Bile acid synthesis includes the classical pathway and alternative pathway mediated by cholesterol 7α-hydroxylase (CYP7A1) and sterol 27-hydroxylase (CYP27A1) respectively, and is modulated by nuclear receptor farnesoid X receptor (FXR)-mediated negative feedback regulation [18, 19]. Moreover, the reabsorption of intestinal bile acids, which enables bile acids to return to the liver and maximizes the use of it through enterohepatic circulation, also has a vital influence on cholesterol homeostasis [20, 21]. Previous studies have clearly revealed that the hypocholesterolemic action of functional foods supplement is closely related to enterohepatic circulation of bile acids [22, 23]. Therefore, it is necessary to consider the regulatory effect of l-arabinose on cholesterol metabolism from the perspective of bile acid metabolism.

Although previous studies have shown the hypocholesterolemia properties of l-arabinose, how l-arabinose improved cholesterol homeostasis via the modulation of bile acid metabolism needs further research. In the present study, hypercholesterolemic mice induced by HFHSD were applied to assess the effect and possible molecular mechanisms of cholesterol-lowering in the regulation of bile acid metabolism after l-arabinose supplementation. The results showed that dietary l-arabinose exhibited effects on alleviating HFHSD-induced hypercholesterolemia through regulating bile acid metabolism.

## Methods

### Materials and chemicals

l-Arabinose was obtained from Sigma-Aldrich (W325512). LDL-C and HDL-C enzymatic reagent kits were purchased from Nanjing Jiancheng Bioengineering Institute (Nanjing, China). LabAssay TG and LabAssay TC were obtained from WAKO (Japan). Total bile acid assay kit was purchased from Huijia Biotechnology (Huijia Biotechnology, China). RNAiso Plus and Prime Script RT system were purchased from Takara Biomedical Technology (Beijing, China). Primary antibodies against 3-hydroxy-3-methylglutaryl-CoA reductase (HMGCR), sterol regulatory element binding protein-1c (SREBP-1c), and bile acid transporter (ASBT) were purchased from Proteintech (IL, USA). Antibodies against CYP7A1, hepatic nuclear factor 4α (HNF-4α), and Ileal-bile acid-binding protein (I-BABP) were purchased from Santa Cruz Biotechnology (CA, USA). Antibodies against CYP27A1 and oxysterol 7α-hydroxylase (CYP7B1) were acquired from Abcam (Cambridge, UK). Antibodies against FXR, GAPDH, and HSP90 were purchased from Cell Signaling Technology (MA, USA).

### Animal

C57BL/6 J male mice (6 weeks old, 18–20 g) were purchased from Shanghai SLAC Laboratory Animal Co., Ltd (Shanghai, China). All mice were kept in specific pathogen-free conditions (24 ± 2 °C, 60% relative humidity, and 12 h light/dark cycle) and given food and water ad libitum. All animal protocols and procedures were performed following the approval from the Laboratory Animal Ethics Committee of Jiangnan University (University JN. No20190315c0320630 [26]). After 1 week of acclimation, thirty mice were randomly divided into the control group, the high-fat-high-sucrose diet-fed group (HFHSD group), and HFHSD fed mice receiving l-arabinose (HFHSD + L-Ara group, Sigma-Aldrich, administrated intragastrically with 400 mg/kg/day l-arabinose for 12 weeks). The selection of dose was based on previous research [6]. The control and HFHSD groups were intragastrically administrated with same volume of distilled water per day. Mice in HFHSD and HFHSD + L-Ara group received a high-fat diet (Research Diets, D12492), accompanied by a 10% glucose solution (Sigma-Aldrich) for 12 weeks. Urine and feces were collected and stored at − 80 °C at the end of the experiments. The body weight of all mice was weekly monitored. Mice were sacrificed and plasma, liver, epididymal fat, and ileum were collected respectively. All tissue samples were frozen in liquid nitrogen and stored at − 80 °C for further analysis.

### Biochemical analysis

Plasma concentrations of LDL-C and HDL-C were determined using enzymatic reagent kits (Nanjing jiancheng, China). The levels of total bile acids of serum, feces, and urine were measured using commercial assay kits (Nanjing jiancheng, China). TC and TG concentrations in the liver were examined according to the manufacturer’s protocols (Wako, Japan).

### Histological analysis

To determine the architecture changes and the size of lipid droplets in the liver, hematoxylin and eosin (H&E) staining were performed as previously described [24]. Briefly, liver tissues were fixed in 10% neutral formalin for 24 h. Then, after being embedded in paraffin and sectioned at 5 μm thickness, tissue sections were stained with H&E. The section images were observed under an inverted light microscope (Axio Vert. A1, Carl Zeiss Microscopy GmbH, Germany).

### Quantitative real-time PCR analysis (qRT-PCR)

Total RNA was isolated from the liver and ileum tissues using RNAiso Plus (Takara, China). cDNA was synthesized using the Prime Script RT system (Takara, China). qRT-PCR was performed using the ABI 7900 RT-PCR system (Applied Biosystem, USA) for the expression levels of genes related to cholesterol and bile acid metabolism. The relative mRNA expression level was normalized to 18S. The sequences of qRT-PCR primers were shown in Additional file 1: Table S1.

### Western blot analysis

Western blot analysis was performed according to the method described previously [25]. Liver or ileum tissues were homogenized using RIPA lysis buffer (Beyotime, Shanghai, China). After complete lysis and collection of the supernatant, the protein concentration was determined using the BCA protein assay reagent (Beyotime, Shanghai, China). 30 μg of each protein sample was separated on 10% SDS-PAGE and then transferred to polyvinylidene fluoride (PVDF) membranes. The membranes were incubated with corresponding primary antibodies overnight at 4 °C. Subsequently, membranes were incubated with appropriate secondary antibodies at room temperature for 1.5 h. The protein expression level was normalized to the HSP90 or GAPDH. The quantification of the protein band intensity was determined by Image J software.

### Statistical analysis

Statistical analysis was performed with GraphPad Prism 8.0 (GraphPad Software). Results were expressed as the mean ± standard error of mean (SEM) with at least three independent experiments. Statistical significance among three groups was analyzed by one-way ANOVA with Tukey’s post hoc test. Statistical significance was defined at *p* < 0.05.

## Results

### l-Arabinose reduces HFHSD-induced body weight gain

The HFHSD significantly increased body weight gain and index of liver and epididymal white adipose tissues compared with the control diet. In contrast to the HFHSD group, the relative weight of liver and epididymal white adipose tissues exhibited a more obvious reduction in l-arabinose-treated mice, which contributed to the higher body weight loss in the l-arabinose group. Furthermore, no significant difference in body weight gain and liver index was observed between the control group and the l-arabinose group. However, the epididymal white adipose tissues index was significantly higher in the l-arabinose group than that of the control group.

### l-Arabinose improves lipid metabolism of HFHSD-fed mice

A significant rise in the serum level of LDL-C and a decline in HDL-C content were observed in the mice fed HFHSD compared with the control group. On the contrary, l-arabinose administration significantly reduced the serum LDL-C content and increased HDL-C level (Fig. 2A). Furthermore, mice in the HFHSD-fed group had significantly higher hepatic TC and TG levels compared with the control group. The suppression of hepatic TC and TG by l-arabinose was also apparently observed (Fig. 2B). In addition, as shown in the H&E staining of the liver tissue, the HFHSD-induced increases in lipid droplets, vacuoles, and disordered arrangement were attenuated by l-arabinose administration (Fig. 2C). The accumulation of hepatic lipids caused by HFHSD can lead to inflammation in the liver [26]. Therefore, the expression of hepatic inflammatory genes was also determined. As displayed in Fig. 2D, the intervention of l-arabinose for 12 weeks inhibited the mRNA level of tumor necrosis factor alpha (TNF-α) and and significantly increased interferon-γ (IFN-γ), interleukin-1β (IL-1β) mRNA levels compared to those of the HFHSD-fed group. No significant change in interleukin-6 (IL-6) mRNA level was observed between the control group and l-arabinose group under the same assay condition. These results indicated that l-arabinose improved lipid metabolism disorder and hepatic steatosis induced by HFHSD.

### l-Arabinose suppresses hepatic cholesterol synthesis and facilitates reverse cholesterol transport

Given that l-arabinose administration could alleviate the elevated serum lipid levels and hepatic lipid accumulation caused by HFHSD, we further investigated whether l-arabinose suppresses hepatic lipids synthesis, especially hepatic cholesterol. The mRNA expressions of the key genes controlling cholesterol metabolism in the liver were measured. As shown in Fig. 3A, the mRNA levels of HMGCR and SREBP-1c significantly increased in HFHSD-fed mice, which mediate the key step in cholesterol and triglycerides synthesis. l-Arabinose treatment significantly downregulated the mRNA expressions of HMGCR and SREBP-1c compared with the HFHSD group. However, l-arabinose treatment slightly increased the mRNA expressions of liver X receptor (LXR) and sterol regulatory element-binding protein-2 (SREBP2) expression but no significant difference was found compared with the HFHSD group. Moreover, similar modulation of the protein expressions of HMGCR and SREBP-1c was observed in mice treated with l-arabinose (Fig. 3B, C). In addition, the expression alternation of genes related to reverse cholesterol transport in the liver, including low-density lipoprotein receptor (LDL-R), scavenger receptor class B type 1 (SR-B1), ATP binding cassette transporter G1 (ABCG1), and ATP binding cassette transporter A1 (ABCA1) were detected. The results showed that l-arabinose intervention significantly increased the mRNA expressions of LDL-R, SR-B1, ABCG1, as well as ABCA1 in comparison with the HFHSD group (Fig. 3D). Taken together, these above results demonstrated that l-arabinose could effectively inhibit cholesterol synthesis and enhance reverse cholesterol transport, thus lowering plasma and hepatic cholesterol levels.

### l-Arabinose promotes the bile acid synthesis in the liver

Cholesterol catabolism to bile acids plays a key role in cholesterol homeostasis since bile acids are the major metabolites of cholesterol. We next determined the effects of l-arabinose on bile acid synthesis. As shown in Fig. 4A, l-arabinose markedly upregulated the mRNA expression of CYP7A1 compared with the HFHSD group, while no significant changes were observed in other key enzymes for classical or alternative bile acid synthesis pathways including CYP27A1, CYP7B1, and sterol 12α-hydroxylase (CYP8B1). Moreover, the protein levels of CYP7A1 and CYP27A1 were elevated in the liver from HFHSD-fed mice treated with l-arabinose (Fig. 4B, C).

Negative feedback regulation plays an important role in bile acid synthesis [27, 28]. The alternation of key genes related to negative feedback of hepatic bile acid synthesis, including FXR, small heterodimer partner (SHP), as well as HNF-4α were presented in Fig. 4D. The mRNA and protein expressions of FXR were downregulated in mice treated with l-arabinose. As the target gene of FXR, SHP was shown to repress bile acid synthesis through inhibition of HNF-4α [29]. Similarly, l-arabinose supplementation also lowered mRNA expression of SHP following FXR in the liver, leading to increased expression of HNF-4α at both protein and mRNA levels (Fig. 4E, F). In addition, bile salt export pump (BSEP) is responsible for hepatic bile acids efflux into canaliculi [18]. The mRNA expression of BSEP was higher in the liver of l-arabinose-treated mice than that of the HFHSD group, which might be in response to increased bile acid synthesis (Fig. 4E).

### l-Arabinose enhances the bile acids excretion and reduces the intestinal bile acid reabsorption

Based on the results above, l-arabinose treatment could enhance the cholesterol uptake from the circulation and conversion to bile acids, so we speculated that l-arabinose might affect the reabsorption and excretion of bile acids. Therefore, we first measured the content of bile acids in the feces and urine of mice. The excretion of bile acids both in the HFHSD and l-arabinose group was significantly higher than that of the control group. Notably, l-arabinose treatment remarkably increased bile acid excretion in comparison with the HFHSD group (Fig. 5A). Meanwhile, l-arabinose intervention slightly increased the content of bile acids in urine (Fig. 5B). In contrast to the HFHSD group, l-arabinose treatment significantly decreased the level of total serum bile acids (Fig. 5C). Besides that, the level of fecal cholesterol was no significant difference among the three groups (Fig. 5D).

In addition, l-arabinose had apparent effects on the reabsorption of bile acids in the small intestine. Regulatory factors involved in the reabsorption of bile acids, including ASBT, I-BABP, and FXR, were downregulated in mice given l-arabinose compared to the HFHSD and control group (Fig. 5E). Consistently, l-arabinose treatment also reduced the protein expressions of ASBT and FXR in the ileum (Fig. 5F, G). The mRNA expression of fibroblast growth factor 15 (FGF15), as an FXR target gene in the intestine, was largely attenuated in mice fed l-arabinose. Besides, l-arabinose had no obvious effect on cholesterol absorption in the ileum (Additional file 1: Fig. S1A). These results revealed that l-arabinose could promote the excretion of fecal bile acids by suppressing the reabsorption of intestinal bile acids.

## Discussion

Hypercholesterolemia and hypertriglyceridemia are the main hallmarks of CVD [1]. Dietary intervention of cholesterol-lowering functional foods of natural origin has attracted much research interest in CVD prevention and treatment [3]. Several lines of evidence suggested that l-arabinose is involved in the improvement of lipid metabolic disorder and immune regulation [5]. Whereas the contributions of l-arabinose to cholesterol metabolism were yet to be sufficiently evaluated. In the present study, hypercholesterolemic mice induced by HFHSD were used to assess the effects of l-arabinose on cholesterol metabolism based on the regulation of bile acid metabolism, and the possible molecular mechanism underlying its hypocholesterolemic activity was preliminarily explored.

**Fig. 1:**
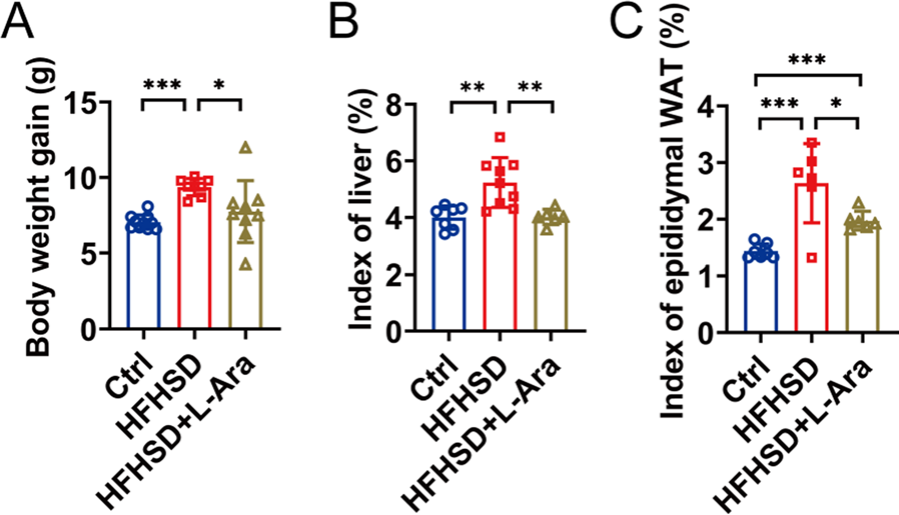
Effects of l-arabinose on body weight gain and index of liver and epididymal white adipose tissues. Body weight gain (**A**), index of liver (**B**), and index of epididymal white adipose tissues (**C**) in HFHSD-fed mice after 12 weeks treatment of l-arabinose. Data are shown as mean ± SEM. *p < 0.05; **p < 0.01; ***p < 0.001. *WAT* white adipose tissues, *Ctrl* control group, *HFHSD* HFHSD-fed group, *HFHSD* + *L-Ara* HFHSD-fed group treated with l-arabinose

**Fig. 2:**
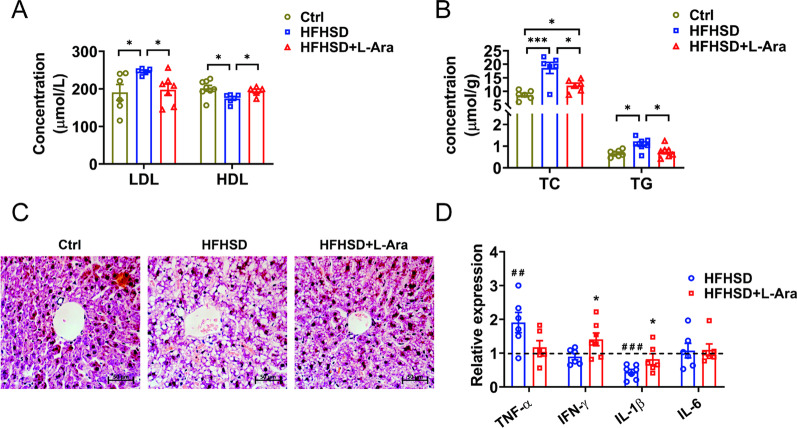
Effects of l-arabinose on lipid levels in the serum and liver of HFHSD-fed mice treated with or without l-arabinose for 12 weeks. Serum HDL-C and LDL-C levels (**A**) and hepatic TC and TG levels (**B**). Representative H&E-stained images of liver sections (magnification, × 400) (**C**). The mRNA expression of hepatic key inflammatory markers, TNF-α, IFN-γ, IL-1β, and IL-6, determined by RT-qPCR analysis (**D**). The mRNA expression levels were normalized to 18S and were shown relative to mice in the control group. Data are shown as mean ± SEM. ^##^*p* < 0.01 compared with the control group; ^###^*p* < 0.001 compared with the control group; *p < 0.05 compared with the HFHSD-fed group. *Ctrl* control group, *HFHSD* HFHSD-fed group, *HFHSD + L-Ara*, HFHSD-fed group treated with l-arabinose

**Fig. 3:**
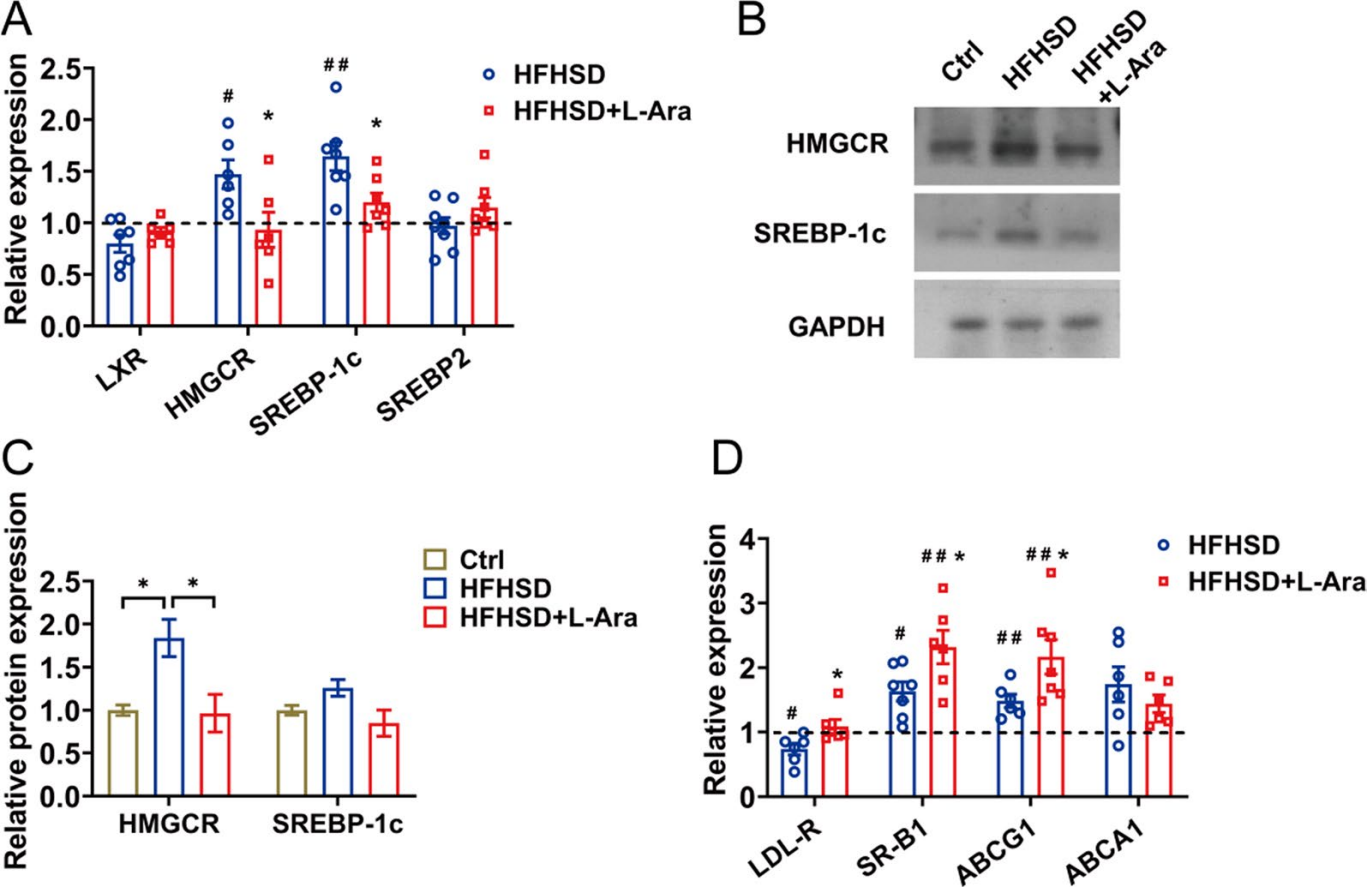
l-Arabinose modulates hepatic cholesterol synthesis and facilitates reverse cholesterol transport. Effects of l-arabinose on the relative expression of LXR, HMGCR, SREBP-1c, and SREBP2 measured by qRT-PCR analysis (**A**) in the liver of HFHSD-fed mice treated with or without l-arabinose for 12 weeks. Effects of l-arabinose on protein levels of SREBP-1c and HMGCR in the liver (**B**) with densitometric quantification (**C**). Expression of key genes in reverse cholesterol transport in the liver (**D**). The mRNA and protein expression levels were normalized to 18S and GAPDH respectively and were shown relative to mice in the control group. Data are shown as mean ± SEM. ^#^*p* < 0.05 compared with the control group; ^##^*p* < 0.01 compared with the control group; *p < 0.05 compared with the HFHSD-fed group. *Ctrl* control group, *HFHSD* HFHSD-fed group, *HFHSD* + *L-Ara* HFHSD-fed group treated with l-arabinose

It has been reported that l-arabinose prevents and improves lipid metabolic disorder in other diet-induced hypercholesterolemia, thereby might play a role in diet-induced hyperlipidemia, atherosclerosis, and CVD [30]. Consistent with previous animal studies [9], mice in the l-arabinose group showed reductions in body weight gain, index of liver and epididymal white adipose compared to those of the HFHSD-fed group, which contributed to the improvement of hypercholesterolemia (Fig. 1). The beneficial effects of l-arabinose on improving serum lipid profiles by effectively lowering serum TC and TG content in HFHSD-fed mice had been displayed in our previous study [31]. As an extension of our previous study, l-arabinose administration also prevented the increases in serum LDL-C, hepatic TC, and TG levels, accompanied by elevated serum HDL-C concentration in this study, indicating l-arabinose exerted cholesterol-lowering effects to some extent (Fig. 2). Furthermore, the accumulation of lipids in the liver caused by consuming HFHSD can give rise to hepatic inflammation [26]. Our observation of regulatory effects of l-arabinose on inflammatory cytokines, by lowering TNF-α and increasing IFN-γ, IL-1β mRNA levels in the liver, suggested that l-arabinose supplementation may improve hepatic inflammation induced by HFHSD. Similar observations on inhibitory effects of l-arabinose on TC and TG levels and inflammation in the liver have been reported before [9].

l-Arabinose supplementation was reported to regulate glucose homeostasis via inhibition of hepatic gluconeogenesis and improvement of insulin sensitivity, thus suppressing elevated plasma glucose and insulin levels in metabolic disorder mice [31]. Since insulin promotes cholesterol synthesis, supplementation of l-arabinose may help disrupt endogenous cholesterol biosynthesis. HMGCR catalyzes the rate-limiting step of cholesterol synthesis, and downregulation of HMGCR at protein and mRNA levels was observed, which might be related to a lower level of hepatic cholesterol in the l-arabinose group (Fig. 3A, B). In addition, our previous work observed that protective effects of l-arabinose on abnormal gluconeogenesis were associated with the activation of AMP-activated protein kinase (AMPK). l-Arabinose promoted the activation of AMPK and reduced acetyl-CoA carboxylase (ACC) activity, which helps to inhibit fatty acid synthesis, subsequently leading to the reduction of hepatic lipid accumulation in metabolic disorder mice caused by HFHSD [31]. Moreover, animal studies also showed that l-arabinose could reduce lipid levels by acting as an inhibitor of liver lipogenic enzymes, such as ACC, ATP citrate-lyase, and fatty acid synthase in rats with high dietary sucrose [30]. Besides, Hao et al. found that l-arabinose dramatically ameliorated metabolic syndrome by upregulating the genes participated in energy expenditure pyruvate dehydrogenase kinase 4 (PDK4) and carnitine palmitoyltransferase 1α (CPT-1α) and downregulating adipogenesis genes ACC [8]. Our studies were also in line with these conclusions.

Concentrations of circulating cholesterol, including LDL-C and HDL-C, were governed by reverse cholesterol transport, in which cholesterol from peripheral tissues is transported to the liver for subsequent hepatic catabolism into bile and excretion [32, 33]. LDL-R and SR-B1 are two key receptors in this process [34]. LDL-R mediates the removal of plasma LDL-C, whereas SR-B1, as the high-affinity receptor of HDL, is responsible for the selective uptake of HDL lipids [35, 36]. Upregulations of LDL-R and SR-B1 expression were observed after l-arabinose treatment, which was associated with decreased plasma cholesterol level (Fig. 3D). Besides, ABCG1 and ABCA1 contribute to the process of reverse cholesterol transport, which facilitates the removal of excess cholesterol from peripheral tissue back to the liver for further metabolism. ABCG1 mainly mediates intracellular cholesterol efflux to HDL, while ABCA1 promotes cholesterol flow to apolipoprotein A1 [37]. Expressions of ABCG1 and ABCA1 were elevated in response to l-arabinose intervention, which in line with the increase in plasma HDL-C levels, therefore preventing excessive cellular lipids accumulation (Fig. 3D). However, the regulatory effects of l-arabinose on the reverse cholesterol transport process in peripheral tissue, especially in macrophages, remain to be explored in our future research. Our data revealed that the cholesterol-lowering effect of l-arabinose may be attributed in part to its role in suppressing cholesterol synthesis and promoting reverse cholesterol transport.

Bile acid reabsorption and cholesterol absorption in the small intestine are closely related to maintaining cholesterol homeostasis, and lowering cholesterol function of other substances is closely linked to these two pathways [23, 38]. For instance, the underlying mechanisms for the hypocholesterolemic activity of β‑sitosterol laurate involve these two pathways mediated by reducing ASBT and I-BABP levels and downregulating intestinal niemann-pick c1-like protein 1 (NPC1L1) respectively, therefore, increasing the excretion of fecal bile acids and cholesterol [39]. We next explored whether l-arabinose maintains cholesterol homeostasis by regulating bile acid reabsorption and cholesterol absorption. Most bile acids are reabsorbed into the intestinal epithelial cells by ASBT mainly localized at the enterocyte brush border, then transported to the basolateral membrane with the assistance of I-BABP, and finally, efflux into the portal blood and transport to the liver [40, 41]. Further research found that the inhibition of bile acid reabsorption by l-arabinose also occurred via depressing the expression of ASBT and I-BABP in the ileum, which was consistent with the results of increasing the fecal bile acids level and lower serum bile acid content in the l-arabinose group (Fig. 5E, F). Besides, the expressions of key regulatory factors related to cholesterol absorption and excretion in the ileum, including NPC1L1, microsomal triacylglycerol transport protein (MTP), ATP binding cassette transporters 5 and 8 (ABCG5 and ABCG8), acyl CoA cholesterol acyltransferase 2 (ACAT2), were determined to explore the effect of l-arabinose on cholesterol absorption. As shown in Additional file 1: Fig. S1A, the less relevance of cholesterol absorption to the hypocholesterolemic effects of l-arabinose was observed as indicated by unchanged expression of related genes, which was consistent with the results of fecal cholesterol content in the l-arabinose group. Furthermore, previous studies have confirmed that l-arabinose could protect the intestinal barrier from dextran sodium sulfate-induced colitis and gliadins-induced damage [6, 42]. In the present study, l-arabinose intervention could increase the mRNA expression of tight junction proteins, mainly including ZO-1, occludin, and claudin, which contributed to maintaining intestinal barrier integrity and epithelial barrier function (Additional file 1: Fig. S1B).

**Fig. 4:**
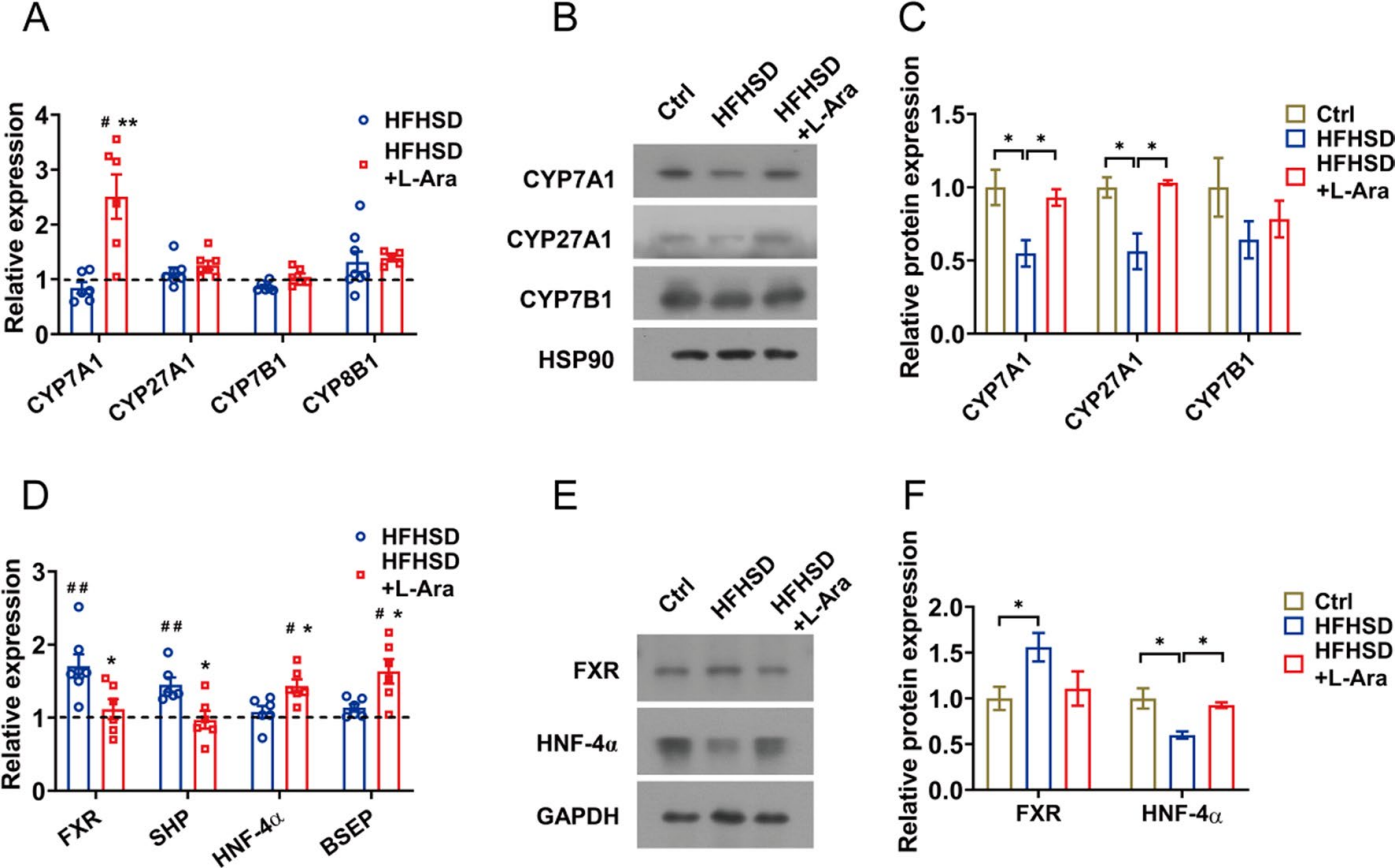
l-Arabinose promotes bile acid synthesis in the liver. The hepatic mRNA expressions of CYP7A1, CYP27A1, CYP7B1, and CYP8B1 (**A**) in HFHSD-fed mice treated with or without l-arabinose for 12 weeks. The protein levels of CYP7A1, CYP27A1, and CYP7B1 in the liver (**B**) with densitometric quantification (**C**). HSP90 was used as an internal reference. The hepatic mRNA expressions of FXR, SHP, HNF-4α, and BSEP (**D**). The protein expression of hepatic FXR and HNF-4α (**E**) with densitometric quantification (**F**).GAPDH was used as an internal reference. Data are shown as mean ± SEM. ^#^*p* < 0.05 compared with the control group; ^##^*p* < 0.01 compared with the control group; *p < 0.05 compared with the HFHSD-fed group; **p < 0.01 compared with the HFHSD-fed group. *Ctrl* control group, *HFHSD* HFHSD-fed group, *HFHSD* + *L-Ara* HFHSD-fed group treated with l-arabinose

**Fig. 5:**
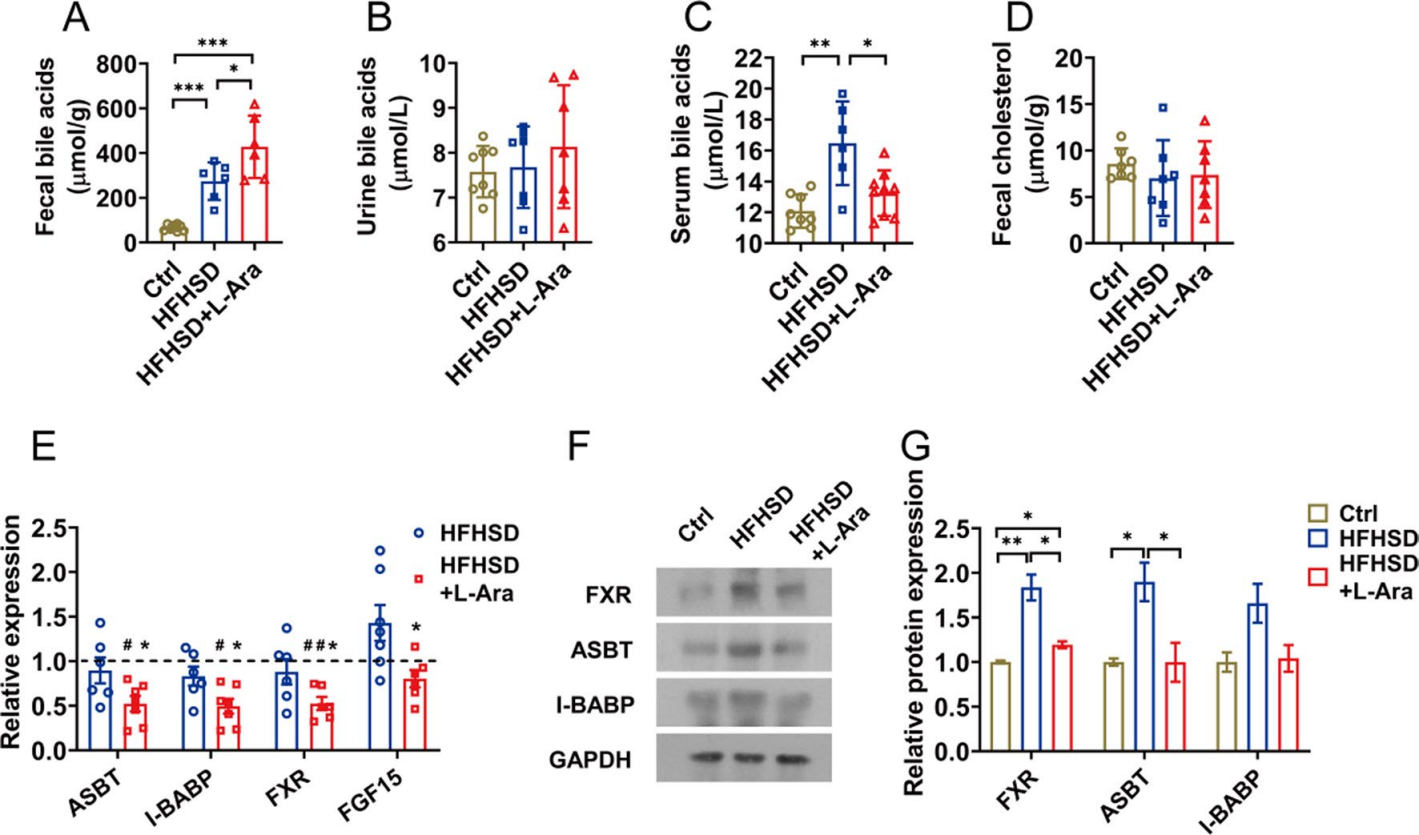
l-Arabinose enhances the bile acids excretion and reduces intestinal bile acids reabsorption. Total bile acids content in the feces (**A**), urine (**B**) and serum (**C**), and fecal cholesterol level (**D**) in HFHSD-fed mice treated with or without l-arabinose for 12 weeks. The ileal mRNA expressions of ASBT, I-BABP, FXR, and FGF15 determined by qRT-PCR analysis (**E**). The ileal protein expressions of FXR, ASBT, and I-BABP determined by western blotting (**F**) with densitometric quantification (**G**). Data are shown as mean ± SEM. ^#^*p* < 0.05 compared with the control group; ^##^*p* < 0.01 compared with the control group; *p < 0.05 compared with the HFHSD-fed group. *Ctrl* control group, *HFHSD* HFHSD-fed group, *HFHSD* + *L-Ara* HFHSD-fed group treated with l-arabinose

**Fig. 6:**
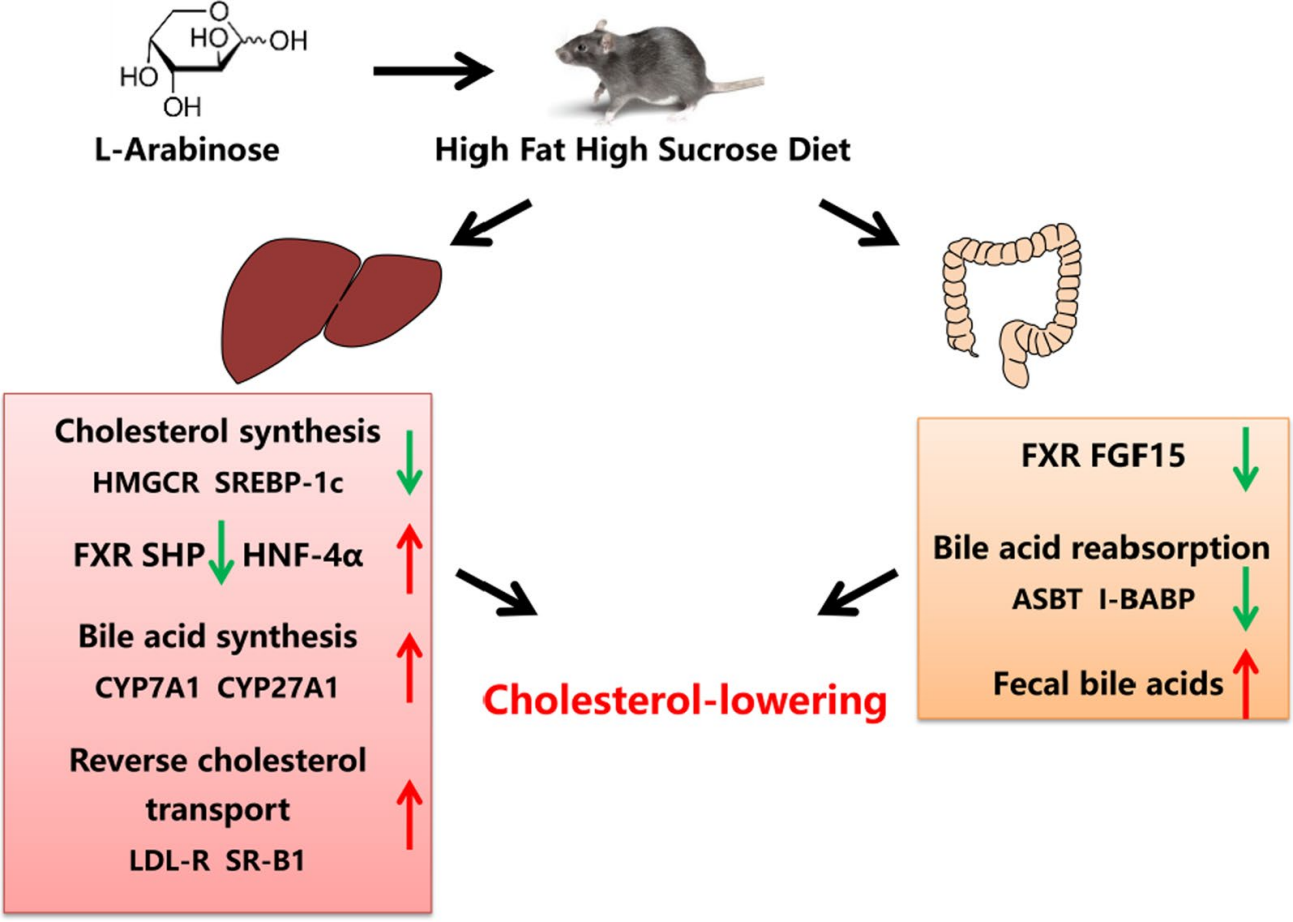
Graphical abstract of the effects of l-arabinose on improving hypercholesterolemia in HFSD-fed mice. l-Arabinose not only inhibited cholesterol synthesis but also facilitated cholesterol transport from peripheral tissue to the liver, so as to reduce circulating cholesterol. Moreover, dietary administration with l-arabinose had beneficial effects on bile acids homeostasis by reducing the reabsorption of bile acids and promoting hepatic bile acid synthesis, thus accelerating the excretion of bile acids while enhancing the decomposition of cholesterol into bile acids, ultimately facilitating cholesterol homeostasis and alleviating hypercholesterolemia. Arrows (↑) represent upregulation of protein or mRNA expression. Arrows (↓) represent downregulation

As the major end product of cholesterol catabolism, reabsorption of bile acid in the small intestine could influence the process of hepatic bile acid synthesis due to enterohepatic circulation, consequently adjusting the homeostasis of hepatic cholesterol [17]. As mentioned above, l-arabinose enhanced the excretion of fecal bile acids by reducing the bile acids reabsorption in the ileum, which might lead to hepatic cholesterol depletion. As expected, the impediment to enterohepatic circulation of bile acids promoted hepatic bile acid synthesis in the l-arabinose group, mainly through the CYP7A1 mediated classical pathway, to maintain the balance of the bile acids pool (Fig. 4A, B). Hepatic CYP7A1 expression was downregulated in insulin-resistant mice [43], and the increase in CYP7A1 expression is consistent with our previous study showing that l-arabinose relieved hepatic insulin-resistant state induced by HFHSD or high sucrose diet (HSD). As a critical regulatory factor for bile acid synthesis and transport, FXR has a vital influence on the enterohepatic circulation of bile acids, thus regulating the homeostasis of hepatic cholesterol [44, 45]. l-Arabinose promoted bile acid synthesis in the present work, and we suspected that negative feedback regulation of bile acids mediated by FXR might be activated. However, as reflected by our results, the expression of FXR in the ileum was decreased accompanied by the lower level of fibroblast growth factor 15 (FGF15). Moreover, the expression of FXR and its target gene, SHP, were also inhibited in the liver with a subsequent increase in expression of CYP7A1 at both mRNA and protein levels after l-arabinose treatment (Fig. 5E, F). Our data supported that the negative regulatory effect of FXR on hepatic bile acid synthesis was reserved by l-arabinose, whereas its exact mechanisms are still needed to be investigated. Several studies identified inactivating FXR-mediated negative feedback mechanism by which cholesterol-lowering functional foods accelerate bile acid synthesis [46, 47]. For instance, Geniposide enhanced the hepatic synthesis of bile acids via FXR-mediated negative feedback inhibition of bile acids, leading to the increase in cholesterol catabolism and reverse cholesterol transport [24]. Consequently, it was speculated that the cholesterol-lowering effect of l-arabinose was in part ascribed to diminishing the reabsorption of intestinal bile acids, and then promoted the conversion of hepatic cholesterol into bile acids (Fig. 6).

## Conclusions

In summary, we confirmed that hypercholesterolemia induced by HFHSD in mice could be ameliorated by l-arabinose dietary intervention. The beneficial effects of l-arabinose on HFHSD-induced hypercholesterolemia were associated with improved cholesterol homeostasis via the modulation of bile acid metabolism. Our work provides insight into the development and application of functional foods containing l-arabinose against hypercholesterolemia, but the regulatory mechanism needs to be further investigated.

## Supplementary Information

**Additional file 1. Table S1**: Primer Sequences. **Fig. S1**: Relative mRNA expression of genes involved in cholesterol metabolism in the small intestine of HFHSD-fed mice treated with or without L-arabinose for 12 weeks (A). The mRNA expression levels of ZO-1, occludin, and claudin in ileum sections (B). Data are shown as mean ± SEM. ^#^p < 0.05 compared with the control group; ^##^p < 0.01 compared with the control group; ^###^p < 0.001 compared with the control group; *p < 0.05 compared with the HFHSD-fed group. Ctrl: control group; HFHSD: HFHSD-fed group; HFHSD+L-Ara: HFHSD-fed group treated with L-arabinose. **Fig. S2**: Serum FFA level in HFHSD-fed mice treated with or without L-arabinose for 12 weeks. **p < 0.01 compared with the HFHSD-fed group. Ctrl: control group; HFHSD: HFHSD-fed group; HFHSD+L-Ara: HFHSD-fed group treated with L-arabinose.

## Data Availability

The datasets underlying this article are available from the corresponding author on reasonable request.

## Abbreviations

ABCA1: ATP binding cassette transporter A1
ABCG1: ATP binding cassette transporter G1
ASBT: Apical sodium-dependent bile salt transporter
BSEP: Bile salt export pump
CVD: Cardiovascular diseases
CYP7A1: Cholesterol 7α-hydroxylase
CYP7B1: Oxysterol 7α-hydroxylase
CYP8B1: Sterol 12α-hydroxylase
CYP27A1: Mitochondrial sterol 27-hydroxylase
FXR: Farnesoid X receptor
GAPDH: Glyceraldehyde-3-phosphate dehydrogenase
HDL-C: High-density lipoprotein cholesterol
HMGCR: 3-Hydroxy-3-methylglutaryl-CoA reductase
HNF-4α: Hepatocyte nuclear factor-4α
HFHSD: High-fat-high-sucrose diet
HSP90: Heat shock protein 90
I-BABP: Ileal-bile acid-binding protein
IFN-γ: Interferon-γ
IL-6: Interleukin-6
IL-1β: Interleukin-1β
LDL-C: Low-density lipoprotein cholesterol
LDL-R: Low density lipoprotein receptor
LRH-1: Liver receptor homolog-1
LXR: Liver X receptor
SHP: Small heterodimer partner
SR-B1: Scavenger receptor class B type 1
SREBP-1c: Sterol regulatory element binding protein-1c
SREBP2: Sterol regulatory element-binding protein-2
TC: Total cholesterol
TG: Triglyceride
TNF-α: Sumor necrosis factor-α
