# Supplementary material for: l-Arabinose improves hypercholesterolemia via regulating bile acid metabolism in high-fat-high-sucrose diet-fed mice
Source: Nutr Metab (Lond). 2022 Apr 15;19:30. doi: 10.1186/s12986-022-00662-8 (PMC9013033; doi:10.1186/s12986-022-00662-8)
Supplement: Supplementary file 1 — Additional file 1. Table S1: Primer Sequences. Fig. S1: Relative mRNA expression of genes involved in cholesterol metabolism in the small intestine of HFHSD-fed mice treated with or without L-arabinose for 12 weeks (A). The mRNA expression levels of ZO-1, occludin, and claudin in ileum sections (B). Data are shown as mean ± SEM. #p < 0.05 compared with the control group; ##p < 0.01 compared with the control group; ###p < 0.001 compared with the control group; *p < 0.05 compared with the HFHSD-fed group. Ctrl: control group; HFHSD: HFHSD-fed group; HFHSD+L-Ara: HFHSD-fed group treated with L-arabinose. Fig. S2: Serum FFA level in HFHSD-fed mice treated with or without L-arabinose for 12 weeks. **p < 0.01 compared with the HFHSD-fed group. Ctrl: control group; HFHSD: HFHSD-fed group; HFHSD+L-Ara: HFHSD-fed group treated with L-arabinose. [file 12986_2022_662_MOESM1_ESM.docx]

**Table S1. Primer Sequences**

| gene | forward primer | reverse primer |
| --- | --- | --- |
| TNF-α | CCCTCACACTCAGATCATCTTCT | GCTACGACGTGGGCTACAG |
| IFN-γ | TCAAGTGGCATAGATGTGGAAGAA | TGGCTCTGCAGGATTTTCATG |
| IL-6 | TAGTCCTTCCTACCCCAATTTCC | TTGGTCCTTAGCCACTCCTTC |
| IL-1β | CATCCAGCTTCAAATCTCGCAG | CACACACCAGCAGGTTATCATC |
| LXR | CTCAATGCCTGATGTTTCTCCT | TCCAACCCTATCCCTAAAGCAA |
| HMGCR | AGCTTGCCCGAATTGTATGTG | TCTGTTGTGAACCATGTGACTTC |
| SREBP-1c | TGACCCGGCTATTCCGTGA | CTGGGCTGAGCAATACAGTTC |
| SREBP2 | CAGGTGCAGACGGTACAGG | CGACCCTTACTGGCACTTGAA |
| LDL-R | TGACTCAGACGAACAAGGCTG | ATCTAGGCAATCTCGGTCTCC |
| SR-B1 | TTTGGAGTGGTAGTAAAAAGGGC | TGACATCAGGGACTCAGAGTAG |
| ABCG1 | GCTTGTTGGCCTCAGTTAAG | GTAGCTCAGGCGTACAGAGAT |
| ABCA1 | TTGGCGCTCAACTTTTACGAA | GAGCGAATGTCCTTCCCCA |
| CYP7A1 | GGGATTGCTGTGGTAGTGAGC | GGTATGGAATCAACCCGTTGTC |
| CYP27A1 | CCAGGCACAGGAGAGTACG | GGGCAAGTGCAGCACATAG |
| CYP7B1 | GGAGCCACGACCCTAGATG | GGAGCCACGACCCTAGATG |
| CYP8B1 | CCTCTGGACAAGGGTTTTGTG | GCACCGTGAAGACATCCCC |
| FXR | GCTTGATGTGCTACAAAAGCTG | CGTGGTGATGGTTGAATGTCC |
| SHP | TGGGTCCCAAGGAGTATGC | GCTCCAAGACTTCACACAGTG |
| HNF-4α | CACGCGGAGGTCAAGCTAC | CCCAGAGATGGGAGAGGTGAT |
| BSEP | CACACAAAGCCCCTACCAGT | CCAGAGGCAGCTATCAGGAC |
| ASBT | GTCTGTCCCCCAAATGCAACT | CACCCCATAGAAAACATCACCA |
| I-BABP | CTTCCAGGAGACGTGATTGAAA | CCTCCGAAGTCTGGTGATAGTTG |
| FGF15 | ACGTCCTTGATGGCAATCG | GAGGACCAAAACGAACGAAATT |
| NPC1L1 | GACATCACCTTCCACCTCTTG | CTGGCATTCGACCCATGTAG |
| ACAT2 | AGACTTGGTGCAATGGACTCGAC | CATAGGGCCCGATCCAACAG |
| MTP | CTACCAGGCCCAACAAGAC | CGCTCAATTTTGCATGTATCC |
| ABCG5 | AGCTCTTCCAACACTTCGAC | TACGTTTCTATTTCCCGCTC |
| ABCG8 | GACCTGGTCCTTCTGATGAC | AGAGACTGTGCCTTCTCCAC |
| ZO-1 | ATAGGAGTGCAAGCAGGGAGA | CCATCTCTTGCTGCCAAACTATC |
| occludin | TGAAAGTCCACCTCCTTACAGA | CCGGATAAAAAGAGTACGCTGG |
| Claudin-1 | GACTGTGGATGTCCTGCGTTTC | CAATTACCATCAAGGCTCGGG |
| 18S | ACCGCAGCTAGGAATAATGGA | CAAATGCTTTCGCTCTGGTC |

**
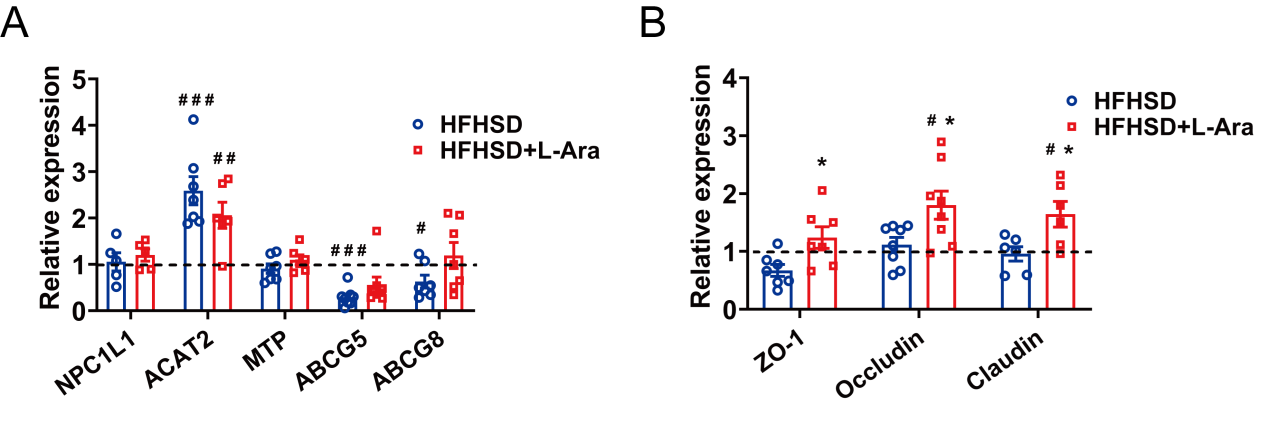
Figure S1.** Relative mRNA expression of genes involved in cholesterol metabolism in the small intestine of HFHSD-fed mice treated with or without L-arabinose for 12 weeks (A). The mRNA expression levels of ZO-1, occludin, and claudin in ileum sections (B). Data are shown as mean ± SEM. ^#^*p* < 0.05 compared with the control group; ^##^*p* < 0.01 compared with the control group; ^###^*p* < 0.001 compared with the control group; **p* < 0.05 compared with the HFHSD-fed group. Ctrl: control group; HFHSD: HFHSD-fed group; HFHSD+L-Ara: HFHSD-fed group treated with L-arabinose.


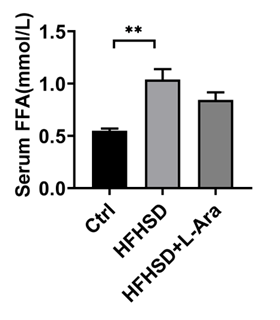


**Figure S2.** Serum FFA level in HFHSD-fed mice treated with or without L-arabinose for 12 weeks. ***p* < 0.01 compared with the HFHSD-fed group. Ctrl: control group; HFHSD: HFHSD-fed group; HFHSD+L-Ara: HFHSD-fed group treated with L-arabinose.
